# Effects of Concentration, Cross-Linking, and Compression on Macromolecular Partitioning in Biological Hydrogels

**DOI:** 10.1021/acsmacrolett.6c00249

**Published:** 2026-07-27

**Authors:** Dan Wang, Juan Pablo Arango Velasquez, Matthew J. Gust, Rishi Dhayananth, Joseph Farroha, Daniel Lee, Nafi Bassoum, Caroline Calodney, Grace Castellano, John Castellano, Saran Diakite, Sena Gebreyesus, Lucas Klingele, Mauricio Lahera-Castillo, Patrick Megahan, Hafsa Musa, Katherine Neviaser, Maxi Ortiz, Priya Rangi, Richard S. Nho, Matthew A. Reilly, Nicholas Ferrell

**Affiliations:** † 12306 The Ohio State University Wexner Medical Center, Department of Internal Medicine, Division of Nephrology, Columbus, Ohio 43201, United States; ‡ 2647 The Ohio State University, Department of Biomedical Engineering, Columbus, Ohio 43201, United States; § 2647 The Ohio State University, Department of Internal Medicine, Division of Pulmonary, Critical Care, and Sleep Medicine, Columbus, Ohio 43201, United States; ∥ 2647 The Ohio State University, Department of Ophthalmology and Visual Sciences, Columbus, Ohio 43201, United States

## Abstract

Transport of macromolecules in biological hydrogels is important for understanding delivery, distribution, and movement of growth factors, cytokines, and peptide- or protein-based drugs within and between biological tissues. This is primarily mediated by basement membranes and extracellular matrixes that separate tissue compartments and structurally support the extracellular microenvironment. Changes in the extracellular matrix (ECM) architecture alter molecular transport and have implications for delivery of biologically active agents in both normal and pathological settings. Matrix density and cross-linking are altered in cancer, fibrotic diseases, and diabetes. Under physiological conditions, ECMs may be compressed to varying degrees under normal or pathological stresses. Here we used multiple biological hydrogels including agarose, collagen I, and Matrigel to understand the relative effects of concentration, cross-linking, and compression on nanoscale molecular transport. We hypothesized that increasing concentration, enzymatic cross-linking, and imparting gel compression would all result in reduced molecular partitioning into hydrogels due to reductions in gel porosity. We found that concentration and compression had significant impacts on molecular partitioning into hydrogels. Cross-linking with multiple enzymatic and chemical cross-linkers increased gel stiffness but had minimal impact on diffusion-based molecular transport. These results indicate that changes in matrix architecture due to increased concentration or reductions in porosity induced by mechanical compression, but not cross-linking, significantly alter macromolecular transport in biological hydrogels.

## Graphical abstract

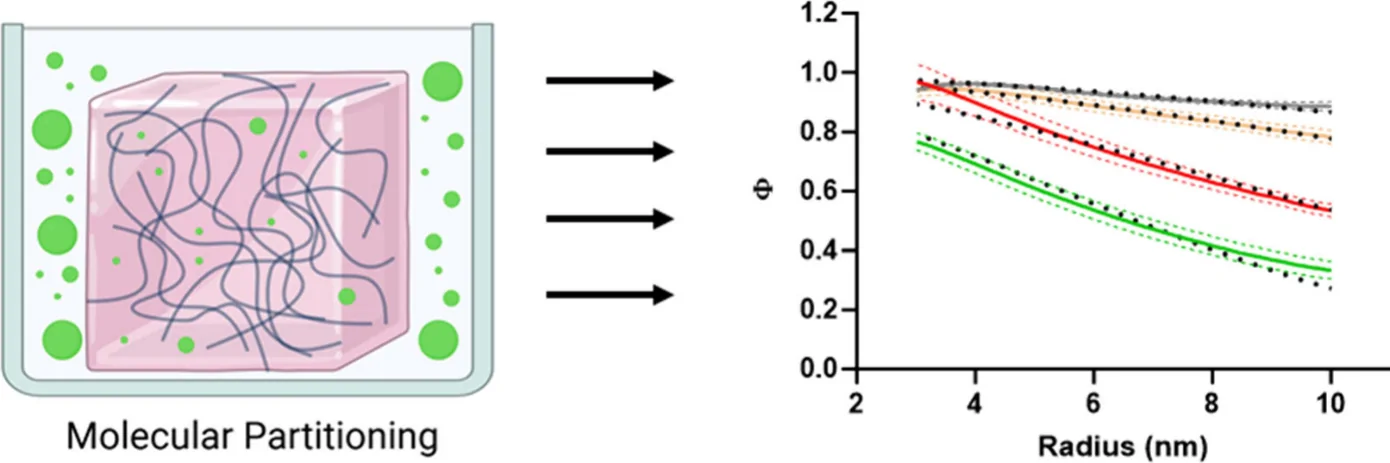

Protein-based hydrogels are ubiquitous in biological tissues. Biological hydrogels play a critical role in providing structural support, regulating cellular function, and controlling fluid and solute transport within and between the tissue compartments. Macromolecular diffusion in porous hydrogels is important in many contexts. The movement of proteins such as growth factors and cytokines through the extracellular matrix (ECM) is critical for biological signaling. Molecular transport through the ECM or across basement membranes regulates movement of biologically active molecules between tissue compartments and has implications for cancer growth and metastasis,^1^ kidney filtration,^2,3^ cartilage function,^4^ vascular permeability,^5,6^ and drug delivery.^7^ Transport phenomena within hydrogels and extracellular matrices are governed by the molecular architecture and charge of the hydrogel, as well as the physical properties of the molecules moving within the gel. Important properties of the hydrogel include its porosity, fiber size, and electrostatic charge. Molecular size, charge, shape, and deformability are important solute properties that govern diffusion. Additionally, the ionic strength of the solvent is important for determining electrostatic interactions.^8–10^ Under normal physiological conditions, many ECMs are subject to mechanical loading including compression or stretch. Examples include articular cartilage, which is subject to repeated loading and unloading, and the kidney, where stretch and compression within the glomerular capillary wall are important for regulating filtration function.^11,12^ Under pathophysiological conditions, the architectural and biophysical properties of the extracellular matrix can be changed. In the setting of cancer and fibrosis, collagen density, alignment, and stiffness are altered.^13,14^ Additionally, the degree of extracellular matrix cross-linking increases in different diseases such as diabetes, cancer, and fibrosis where both enzymatic and nonenzymatic cross-linking mechanisms can be upregulated.^15–18^

Several biophysical parameters have been evaluated with regard to their effects on molecular diffusion in hydrogels. Solute size and gel porosity are critical factors determining the molecular diffusion within hydrogels. The diffusivity will decrease as a function of increasing molecular radius. Additional factors such as solute shape, flexibility, and charge are also important.^9,10^ For example, dextrans of a given hydrodynamic radius exhibit increased molecular partitioning as compared to Ficoll owing to the more spherical and rigid configuration of Ficoll molecules as compared to the linear and flexible nature of dextrans.^19^ Ficoll has also been shown to be more permeable than globular proteins, indicating that Ficoll may also exhibit some molecular flexibility or nonspherical geometry, allowing it to be hyperpermeable as compared to globular proteins such as albumin.^20^ However, this could also be related to the negative charge of proteins as compared to neutral dextran and Ficoll.^21,22^ These phenomena illustrate the complexities of molecular diffusion within biological gels, where multiple properties of the hydrogel, solute, and solvent are relevant to the overall molecular transport within a given system.

The goal of this study was to evaluate how the structural and biophysical properties of hydrogels regulate Ficoll partitioning into multiple biological hydrogels. The relative effects of concentration, compression, and cross-linking on molecular transport within agarose, collagen I gels, and Matrigel were evaluated. We then employed mathematical models of molecular transport into random fiber matrixes to provide insight into the physical properties of the gels. Collagen, Matrigel, and agarose gels have been widely utilized both for modeling transport in biological systems^1,2,10,23–25^ and for in vitro analysis of cell behavior in three-dimensional settings.^26,27^ ECM concentration, degree of mechanical compression, and extent of post-translational cross-linking can change in different biological settings or in disease. We hypothesized that each of these factors would significantly impact molecular partitioning into the hydrogels due primarily to changes in the hydrogel architecture and porosity. As expected, we found that increasing hydrogel concentration reduced the molecular partition coefficient of Ficoll in both agarose and collagen hydrogels in a concentration-dependent manner. In the setting of applied compression, agarose gels exhibited poroelastic behavior, and pore size and molecular partition coefficient were reduced. Cross-linking of collagen and Matrigel with both enzymatic and chemical cross-linkers did not have a significant impact on molecular partitioning into the hydrogels, possibly indicating that cross-linking in ECM gels is primarily intramolecular rather than intermolecular, thus minimizing effects of cross-linking on gel fiber radius and pore size. Alternatively, cross-links could be sufficiently small relative to the ECM fiber radius to minimize the impact of cross-linking on the overall pore structure. Overall, these data suggest that diffusive molecular transport in fibrous biopolymers is primarily mediated by fiber concentration and dynamic changes in hydrogel porosity resulting from mechanical compression. Cross-linking had a significant impact on the mechanical properties of hydrogels, but impact on molecular transport was minimal under the conditions tested in this study.

Agarose gels were fabricated by mixing ultrapure agarose with phosphate-buffered saline (PBS) at varying concentrations (Figure 1) ranging from 0.5 to 4% (w/v). The mixture was heated until it liquefied, poured into a Petri dish, and allowed to cool to room temperature. After the mixture was cooled, cylindrical gels of the appropriate diameter were made with a leather punch. Gel volumes were determined either using the gel mass and accounting for the liquid density (1 g/mL) and agarose density (1.64 g/mL) or by measuring the thickness and diameter with calipers.

**Figure 1:**
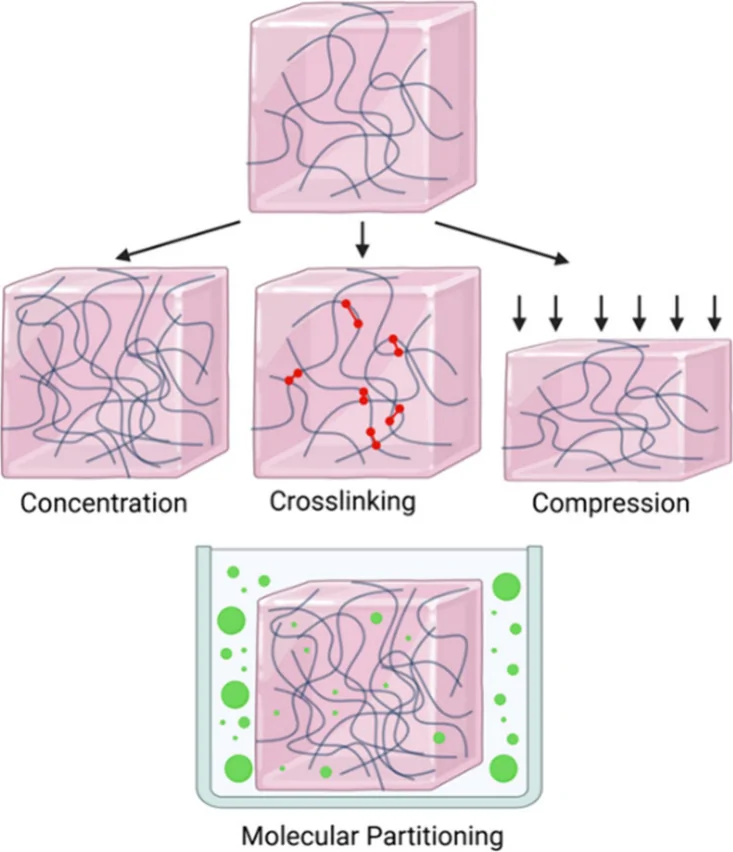
Schematic of approach to measure effects of concentration, cross-linking, and compression on size-dependent molecular partitioning into hydrogels.

ECM hydrogels were made from rat tail collagen I and high concentration Matrigel using standard techniques. Collagen I gels were prepared at concentrations of 2.5 and 9 mg/mL by mixing high concentration rat tail collagen I (≈10 mg/mL, Corning, 354249) neutralized and diluted to the working concentration with appropriate volumes of sterile 10x PBS, 1 M NaOH, and H_2_0 on ice. Collagen gels were transferred to 1.5 mL centrifuge tubes at approximately 100–200 μL sample volumes. Sample volumes were estimated based on the mass of the collagen I gel using an analytical balance. Neutralized collagen was gelled at 37 °C for 30–60 min. Full strength high concentration Matrigel (10.1 mg/mL, Corning, CLS354262) was pipetted using precooled tips into 1.5 mL centrifuge tubes and gelled at 37 °C for 30 min.

ECM gels were cross-linked using chemical and enzymatic cross-linking (Figure 1). For chemical cross-linking, collagen I gels were incubated overnight in 2.5% glutaraldehyde. Gels were then washed three times with an excess volume of PBS. For enzymatic cross-linking, gels were incubated overnight in 100 μg/mL microbial transglutaminase (TG) (Ajinomoto) in PBS. TG was isolated by ion exchange chromatography and purified as described previously.^12^ Gels were washed extensively with PBS to remove any residual enzyme.

Dynamic shear rheometry was used to evaluate the effects of cross-linking on the mechanical behavior of the collagen I gels using a Netzsch ultra+ rheometer (Netzsch Instruments; Burlington, MA). Samples were carefully placed on the rheometer stage and dabbed dry to prevent slippage. A thermal cover was placed to prevent drying. A 20 mm diameter stainless steel upper plate was used. The sample was heated to 37 °C and allowed to equilibrate for 5 min prior to testing. Oscillatory shear was applied with a magnitude of 0.5% strain while decreasing the frequency from 10 to 0.1 Hz. The storage (elastic) shear modulus at 1 Hz was chosen as a metric to compare between groups.

Fluorescently labeled Ficoll was prepared as described previously.^22^ Briefly, Ficoll 70 (Sigma) was heated with fluorescein isothiocyanate (FITC) in the presence of sodium bicarbonate. FITC-Ficoll 70 (referred to further as Ficoll) was precipitated in ethanol, dissolved in water, and purified on a PD-10 gravity flow size exclusion column (GE) to remove unbound FITC. Ficoll stock was stored at 4 °C until use.

The partition coefficient (Φ) was determined by incubating agarose, collagen I, or Matrigel in a dilute solution (1 mg/mL) of Ficoll for 48–72 h to allow for equilibration of Ficoll concentration inside and outside the gel. Previous analysis shows that this time is sufficient to reach equilibrium for similarly sized macromolecules in agarose gels.^25^ By mass balance under these conditions, the partition coefficient can be described by eq 1,^25^

1 $$\Phi =\frac{V_{s}}{V_{g}}\left[\frac{\rho \left(0\right)}{\rho \left(\infty \right)}-1\right]$$

where *V*_s_ and *V*_g_ are the volume of the solute solution and gel, respectively, and ρ(0) and ρ(∞) are the Ficoll concentration in the initial stock solution and the concentration of the solution outside the agarose gel after equilibration. The stock solution and an aliquot of the solution after equilibration were collected and stored at 4 °C until analyzed by high performance liquid chromatography (HPLC). All samples were diluted by 10× in PBS prior to analysis. For collagen and Matrigel, it was difficult to accurately determine the gel volumes. In certain instances, this resulted in partition coefficients for small solutes that were greater than one, which is not physically possible. To account for this, it was assumed that the partition coefficient converged to one at the smallest molecular weights corresponding to the largest measured partition coefficient and the curves were adjusted accordingly.

The Ficoll concentration versus molecular radius was analyzed by size exclusion chromatography (SEC) as described previously.^28,29^ Briefly, Ficoll was analyzed on an Agilent 1260 Infinity II HPLC systems using a Ultrahydrogel 500 column (PSS831913, Waters) at a flow rate of 0.5 mL/min. The mobile phase was phosphate buffer (50 mM phosphate, 150 mM NaCl, 0.01% sodium azide at pH 7.0). Mobile phase was filtered through a 0.22 μm filter. The injection volume was 10 μL per sample. Fluorescence intensity versus elution time was analyzed using a fluorescence detector (G7121A, Agilent) at excitation/emission of 495/520 nm. Ficoll concentration versus molecular radius was determined from the precalibrated column using a custom Matlab program. The size range (hydrodynamic radius) for Ficoll 70 is approximately 2–11 nm.^30^ This corresponds to a molecular weight (MW) range of approximately 10–400 kDa. The relationship between radius (*r*_s_, Å) and molecular weight (Da) is described by *r*_s_ = 0.421MW^0.427^ according to Oliver et al.^31^

The agarose partition coefficient was modeled using the relationship developed by Ogston for partitioning of dilute spherical solutes into a random array of cylindrical fibers according to eq 2.^10,32^

2 $$\Phi =\exp\left[-{\phi \left(1+\frac{r_{s}}{r_{f}}\right)}^{2}\right]$$

where ϕ is the fiber volume fraction, *r*_s_ is the solute radius, and *r*_f_ is the agarose fiber radius. The fiber volume fraction was determined as described according to Johnson et al.,^10^ where ϕ = *C*_a_/ρ_a_ω_a_, where *C*_a_ is the agarose concentration (wt/vol), ρ_a_ is the density of agarose which was taken as 1.64 g/mL,^33^ and ω_a_ is the mass fraction of agarose in the fiber which was taken as 0.625. Based on this calculation, volume fiber fractions for 0.5, 1, 2, and 4% gels (wt/vol) are 0.00488, 0.00976, 0.0195, and 0.0390, respectively, values very similar to the concentration. The experimental data were fit to eq 2 by least-squares error minimization in Excel. The fiber radius was the fit parameter. The pore size of the gels was then estimated using the expression of Giddings et al. that describes the partition coefficient as a function of ϕ, *r*_s_, and the pore radius (*r*_p_) according to eq 3.^34,35^ We estimated the pore size using the molecular radius range from 8 to 10 nm since the model becomes unreliable as the partition coefficient approaches 1.

3 $$\Phi =\left(1-\phi \right){\left(1-\frac{r_{s}}{r_{p}}\right)}^{2}$$

The partition coefficient for collagen I gels and Matrigel were modeled using the expression described by eq 4.^36–38^

4 $$\Phi =\exp-\left[\pi L{\left(r_{f}+r_{s}\right)}^{2}\right]$$

where *L* (cm/cm^3^) is the fiber length per unit of gel volume. The fiber radius for collagen was taken as 9.1 × 10^–8^ cm as described by Shaw and Schy.^24^ The gel fiber length (*L*) was then fit to the experimental data by least-squares error analysis. Matrigel was modeled using the same expression with fiber radius taken as 6.0 × 10^–8^ cm based on the average radius for collagen IV, laminin, and HSPG fibers as described previously.^39^

To compress agarose, gels were placed between two flat plates. Screws were tightened to compress the gels by approximately 30% on the basis of the reduction in their original height. The change in the gel volume induced by compression was determined by measuring the geometry of the gel before and after compression. Compressed gels were then incubated in Ficoll to determine the partition coefficient in the compressed state. The compressed volume was used to calculate the partition coefficient according to eq 1.

Data are shown as the mean ± standard error of the mean (SEM) unless otherwise noted. Statistical analysis was determined by one-way analysis of variance (ANOVA) with posthoc Tukey testing for multiple comparisons or *t* test where appropriate. A *p*-value < 0.05 was considered statistically significant. All analysis was performed in GraphPad Prism.

The Ficoll partition coefficient was calculated using eq 1 for agarose gels of 0.5, 1, 2, and 4 wt %/vol. This analysis showed that for low concentration gels (0.5%), the partition coefficient was near 1 (0.94 ± 0.01) for small solutes (3 nm) and exhibited only weak size dependence with partition coefficient at 10 nm of 0.89 ± 0.02 (Figure 2A). For 1% and 2% gels, partition coefficient at low molecular radii were also near 1, but exhibited more distinct size dependent reductions in partition coefficient with values of 0.78 ± 0.02 and 0.52 ± 0.06 for 1% and 2% gels at 10 nm molecular radius, respectively. The highest concentration agarose gel (4%) had a partition coefficient less than 1 at small molecular radius (3 nm) and exhibited a stronger trend in reduction in the partition coefficient as a function of increased molecular size.

**Figure 2:**
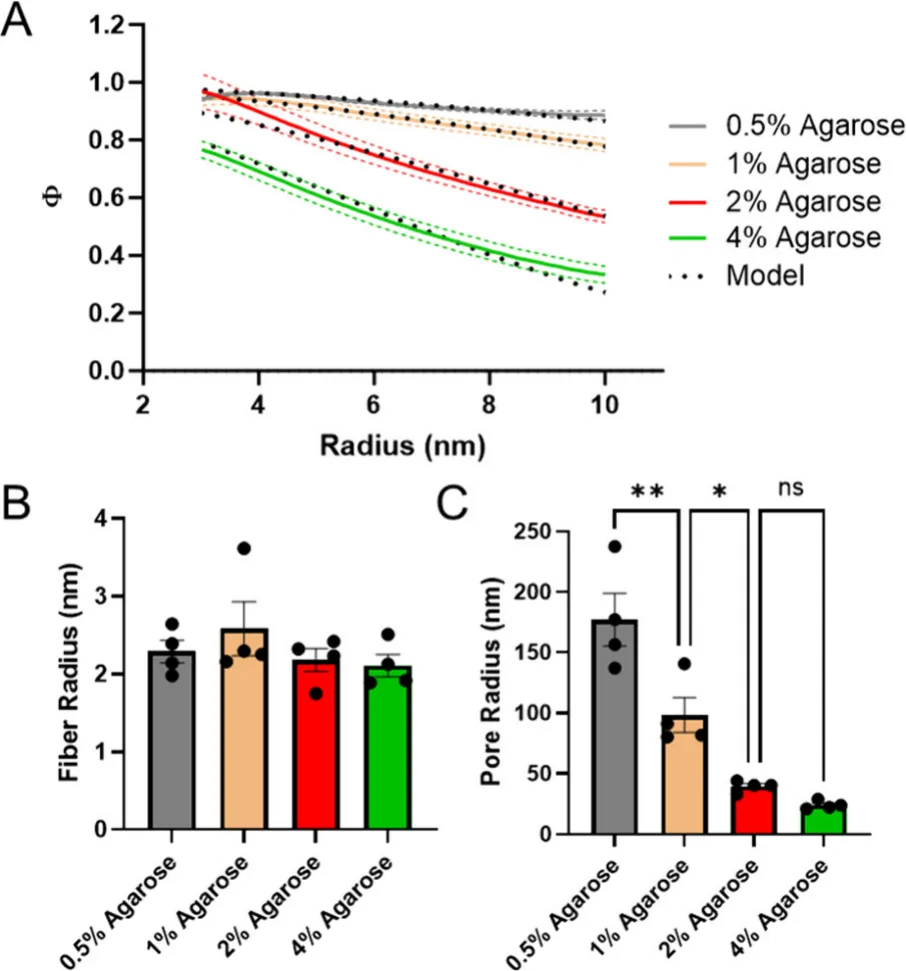
Ficoll partitioning into agarose hydrogels. (A) Ficoll partition coefficient (Φ) vs Ficoll molecular radius for 0.5, 1, 2, and 4% agarose gels. Modeling of the partition coefficient using eq 2 fit well with the experimental data. (B) The fiber radius showed no significant impact of gel concentration. (C) Pore radius showed a significant decrease in pore size with increasing agarose concentration (*n* = 4 per condition, *p < 0.05, **p < 0.01, ns = not significant).

Fitting the experimental data to the mathematical model (eq 2) gave an excellent fit to the data (dashed black lines in Figure 2A). The fiber radius determined based on the best fit to the experimentally measured partition coefficient was approximately 2 nm (Figure 2B). As expected, the fiber radius did not change significantly with increasing gel concentration. The pore size was then determined from eq 3. The pore size for the lowest concentration gel was 177 ± 43 nm and decreased with increasing agarose concentration to 98 ± 29 nm, 39 ± 5 nm, and 24 ± 3 nm for the 1, 2, and 4% gels, respectively (Figure 2C).

The effects of gel concentration on molecular transport into agarose largely conformed to expectations and were consistent with previous studies that analyzed solute transport in agarose gels. The calculated fiber radius was similar to those reported previously, which ranged from 1.6 to 3.3 nm for agarose.^25,33,35^ With regard to the pore radius, the values obtained here were also in the range of those found in previous studies, particularly at low agarose concentrations, and were slightly smaller than those determined previously for higher concentrations of agarose.^35,40^ Overall, these data largely recapitulate the effects of the concentration on size-dependent molecular partitioning into agarose hydrogels. These data show that solute size and gel concentration are the primary factors influencing molecular partitioning into agarose gels, and gel concentration had no significant impact on the fiber geometry but significantly impacted gel porosity.

Effects of concentration on partitioning into collagen gels were then measured in low (2.5 mg/mL) and high concentration (9 mg/mL) gels (Figure 3A). Similar to low-concentration agarose gels, 2.5 mg/mL collagen gels allowed for near-complete partitioning over the full range of molecular sizes evaluated. This is indicated by the essentially flat line (green in Figure 3A) with a very weak size-dependent reduction in partition coefficient with increasing molecular size. A significant size-dependent reduction in partition coefficient was measured in high-concentration (9 mg/mL) gels. The partition coefficient for collagen gels was fit to eq 4 with the fiber length per unit volume (L) as the fit parameter. There was a strong fit to the experimental data for both low- and high-concentration gels. As expected, the total fiber length per unit volume increased with increasing collagen concentration and gave a best fit value of 1.34 × 10^10^ cm/cm^3^ for 2.5% collagen gels and 1.60 × 10^11^ cm/cm^3^ for 9 mg/mL collagen gels.

**Figure 3:**
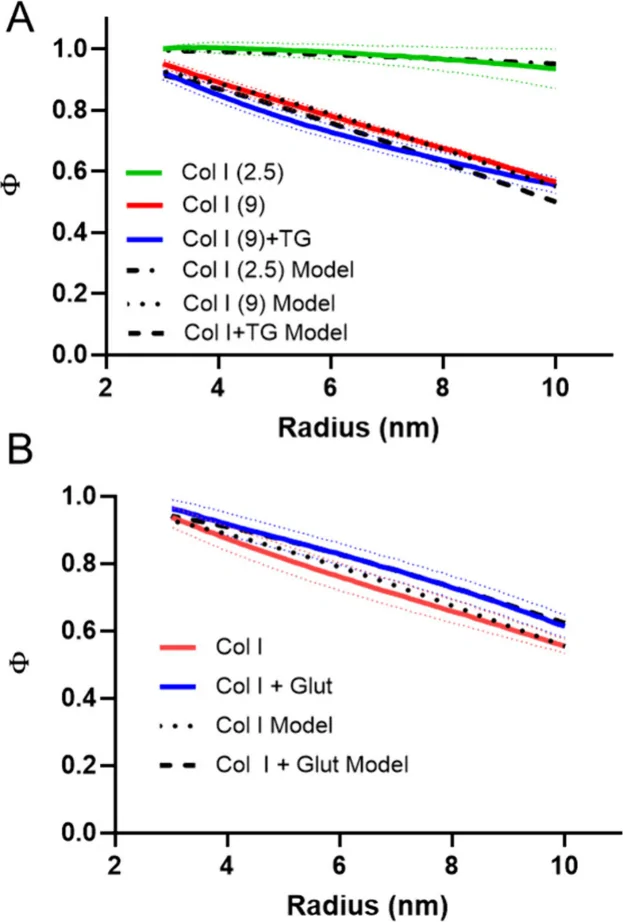
Molecular partitioning into collagen gels with and without enzymatic (transglutaminase, TG) and chemical (glutaraldehyde, Glut) cross-linking. Lower concentration gels showed little size dependent partitioning. At higher concentrations, the partition coefficient showed a size dependent reduction as a function of increasing molecular size. Neither (A) TG or (B) Glut cross-linking significantly altered molecular partitioning into collagen I gels (*n* = 3–4 replicates per condition).

To evaluate effects of cross-linking on molecular partitioning into hydrogels, high concentration collagen gels (9 mg/mL) were utilized. Collagen I can be readily modified using chemical or enzymatic cross-linkers. After cross-linking with either glutaraldehyde or TG, mechanical analysis was performed to confirm that the cross-linking modified the mechanical properties of the collagen gels. Unmodified control gels had a storage modulus of 438 ± 87 Pa (mean ± SEM, *n* = 6), glutaraldehyde cross-linked gels had a modulus of 5396 ± 1678 Pa (*n* = 4), and TG modified gels had a modulus of 11839 ± 5423 Pa (*n* = 5). For both TG and glutaraldehyde modifications, there was minimal impact on the measured partition coefficient (Figure 3A,B). Separate cohorts of unmodified collagen gels (9 mg/mL) were used to compare TG and glutaraldehyde cross-linking as shown by the red lines in Figure 3A,B. Partitioning behavior was similar between control replicates and the fit parameter for the model gave a best fit value of fiber length of 1.60 × 10^–11^ cm/cm^3^ for the first set of control samples (Figure 3A) and 1.69 × 10^–11^ cm/cm^3^ for the second set of native 9 mg/mL collagen gels (Figure 3B). The fiber length for cross-linked collagen gels was similar to control gels with values for TG and glutaraldehyde cross-linked gels giving a best fit value of 1.85 × 10^–11^ cm/cm^3^ and 1.27 × 10^–11^ cm/cm^3^ for TG and glutaraldehyde cross-linked gels, respectively. Glutaraldehyde cross-linked scaffolds had a slightly higher mean partition coefficient compared to native collagen I (Figure 3B). This could be attributed to experimental variability or could be related to changes in gel swelling in modified collagen gels which show reduced swelling following glutaraldehyde cross-linking.^41^ Overall, these data show a strong dependence of concentration on molecular partitioning into collagen I gels, but minimal effect of cross-linking on molecular partitioning into high concentration collagen I gels despite modification of the mechanical properties of the gel.

To further evaluate effects of cross-linking on gel partitioning, a different biopolymer gel system (Matrigel) was utilized. Unmodified Matrigel showed a strong size-dependent reduction in partition coefficient with increasing molecular radius (red line, Figure 4). Similar to collagen I gels, modification of Matrigel with TG did not have any measurable effects on the partition coefficient (blue line, Figure 4). Modeling the partition coefficient using eq 4 gave a best fit value for fiber length (L) for unmodified Matrigel of 3.29 × 10^–11^ cm/cm^3^ and 3.48 × 10^–11^ cm/cm^3^ for TG-modified Matrigel. These data show that, similar to collagen I gels, cross-linking of Matrigel had minimal effects on partitioning into the gels at cross-linker concentrations evaluated here.

**Figure 4:**
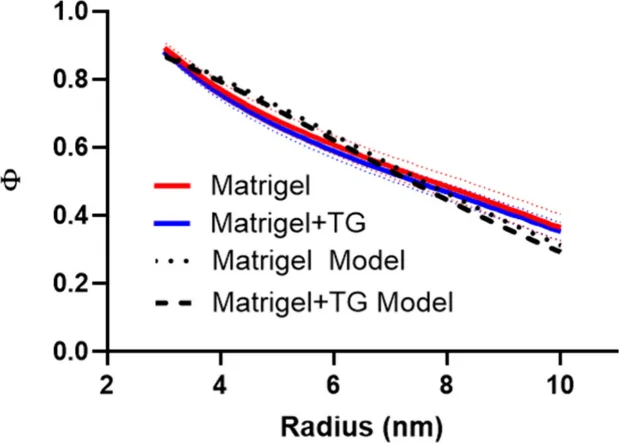
Molecular partitioning into Matrigel with and without transglutaminase cross-linking. TG cross-linking did not significantly affect Ficoll partitioning in Matrigel (*n* = 4 replicates per condition).

Two different cross-linking methods were used to evaluate effects on molecular partitioning. Glutaraldehyde cross-linking is commonly utilized to reinforce collagen and other biopolymer gels and increases the elastic modulus of biopolymer gels.^42,43^ Transglutaminases are enzymes that catalyze glutamine to lysine cross-linking and play a role in both normal physiological matrix cross-linking as well as pathological processes such as fibrosis.^44,45^ Transglutaminases have been utilized for biomaterials development for reinforcing biological gels for various applications including microfluidic cell culture,^46^ promoting tissue repair,^47^ and for reinforcing tissue engineering constructs.^48,49^ Interestingly, cross-linking of different hydrogel systems including collagen gels with either chemical cross-linkers or TG has been shown to minimally effect hydrogel porosity,^23,50,51^ consistent with our data on molecular partitioning into cross-linked collagen I gels and Matrigel.

We then used the agarose gels that showed intermediate size-dependent partitioning (2 wt %/vol) to determine if mechanically compressing the gel would impact molecular partitioning due to poroelastic compression and consequent reductions in pore size. The Ficoll partition coefficient in the compressed at uncompressed agarose gels is shown in Figure 5. Compression resulted in a reduction in the partition coefficient across the range of molecular radii (3–10 nm). This suggests a relatively uniform reduction in pore size leading to reduced partitioning of molecules across the range of molecular sizes. A separate set of control (uncompressed) 2% agarose gels were evaluated for direct comparison with the compressed gels. This analysis showed that the partitioning behavior was very similar between replicate experiments with the 2% gels giving a best fit value (eq 2) for fiber radius of 2.06 ± 0.16 nm compared to 2.18 ± 0.15 for the previous data (Figure 2B) and the pore radius of 37.8 ± 2.4 nm (Figure 5B) compared to 39.4 ± 2.3 nm for the previous experiment shown in Figure 2C. For the compressed gels, it was assumed that changes in gel structure were due to reduced pore size which effectively increases the gel concentration and was not due to changes in the fiber radius. As such, the fiber radius was assumed to be constant at 2.06 nm and the volume fiber fraction was fit to the experimental data using eq 2. The reduction in pore size was then estimated using eq 3 as described previously. This analysis resulted in a statistically significant reduction in the pore radius from 37.8 ± 2.4 nm for the uncompressed gels to 20.2 ± 1.1 nm (Figure 5B). These data demonstrate significant impact of mechanical compression on diffusion of macromolecules over the range of molecular sizes evaluated in this study.

**Figure 5:**
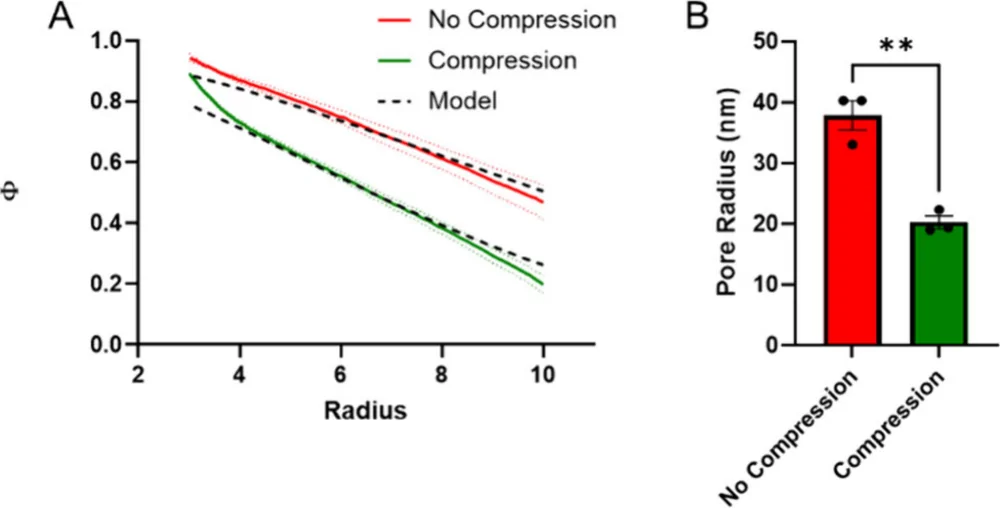
Effects of mechanical compression on partitioning of macromolecules into 2% agarose gels. Gel compression reduced molecular partition coefficient and effective pore size.

The reduction in pore size induced by compression was more pronounced than would be expected if there were a simple linear relationship between the degree of compression and the reduction in pore size. However, Mollenkopf et al. measured the change in water (Darcy) permeability of fibrous gels (fibrin and collagen I) during compression and observed several interesting behaviors.^52^ First, there was a nonlinear decrease in Darcy permeability with increased compression that was particularly pronounced at intermediate strain ranges (10–60%). For collagen gels at 30% strain, there was >50% reduction in permeability. They further observed that under compression, the changes in gel density (porosity) were not uniformly distributed throughout the gel, but rather there were regions of more pronounced density increases near the top of the gel. Particularly, the nonlinear decrease in permeability at similar strain ranges to those tested in this study may partially explain the more significant reduction in molecular permeability compared to what would be expected if compression–permeability relationships were linear and uniform.

In this study, we evaluated how different physical modifications of biological hydrogels impact nanoscale molecular partitioning. Our hypothesis was that increases in gel concentration, cross-linking, and compression would all reduce molecular partitioning by altering the pore structure of the hydrogels. From the measured size-dependent partition coefficient after gel modifications, we conclude that concentration and compression significantly impact molecular diffusion into different biopolymer gel systems. However, cross-linking either by chemical or enzymatic modification had little impact on molecular partitioning into protein-based ECM hydrogels. These data have physiological and pathological implications for understanding how macromolecules move within and between tissue compartments in response to physiologically relevant gel modifications. This could have consequences for growth factor and cytokine transport in different pathological settings such as fibrosis, cancer, and other pathological conditions characterized by changes in ECM architecture, cross-linking, or altered biophysical conditions such as increased mechanical compression. This could further be consequential for protein- and peptide-based drug delivery.

## Abbreviations

ECM: extracellular matrix
TG: transglutaminase
FITC: fluorescein isothiocyanate
PBS: phosphate buffered saline

## References

[1] Ramanujan S., Pluen A., McKee T. D., Brown E. B., Boucher Y., Jain R. K. (2002). Diffusion and convection
in collagen gels: implications
for transport in the tumor interstitium. Biophys.
J..

[2] Fissell W. H., Hofmann C. L., Ferrell N., Schnell L., Dubnisheva A., Zydney A. L., Yurchenco P. D., Roy S. (2009). Solute partitioning
and filtration by extracellular matrices. Am.
J. Physiol. Renal Physiol..

[3] Ferrell N., Cameron K. O., Groszek J. J., Hofmann C. L., Li L., Smith R. A., Bian A., Shintani A., Zydney A. L., Fissell W. H. (2013). Effects of pressure
and electrical charge on macromolecular
transport across bovine lens basement membrane. Biophys. J..

[4] Maroudas A. (1970). Distribution
and diffusion of solutes in articular cartilage. Biophys. J..

[5] Thomsen M. S., Routhe L. J., Moos T. (2017). The vascular basement
membrane in
the healthy and pathological brain. J. Cereb.
Blood Flow Metab..

[6] Michel C., Curry F. (1999). Microvascular permeability. Physiol. Rev..

[7] Brandl F., Kastner F., Gschwind R. M., Blunk T., Teßmar J., Göpferich A. (2010). Hydrogel-based drug delivery systems: Comparison of
drug diffusivity and release kinetics. J. Controlled
Release.

[8] Sassi A. P., Shaw A. J., Han S. M., Blanch H. W., Prausnitz J. M. (1996). Partitioning
of proteins and small biomolecules in temperature-and pH-sensitive
hydrogels. Polymer.

[9] Fatin-Rouge N., Milon A., Buffle J., Goulet R. R., Tessier A. (2003). Diffusion
and partitioning of solutes in agarose hydrogels: the relative influence
of electrostatic and specific interactions. J. Phys. Chem. B.

[10] Johnson E. M., Berk D. A., Jain R. K., Deen W. M. (1995). Diffusion and partitioning
of proteins in charged agarose gels. Biophys.
J..

[11] Butt L., Unnersjö-Jess D., Höhne M., Edwards A., Binz-Lotter J., Reilly D., Hahnfeldt R., Ziegler V., Fremter K., Rinschen M. M. (2020). A molecular mechanism explaining albuminuria
in kidney disease. Nat. Metab..

[12] Wang D., Ferrell N. (2024). Transglutaminase-mediated
stiffening of the glomerular
basement membrane mitigates pressure-induced reductions in molecular
sieving coefficient by reducing compression. Matrix Biol..

[13] Liu F., Mih J. D., Shea B. S., Kho A. T., Sharif A. S., Tager A. M., Tschumperlin D. J. (2010). Feedback amplification of fibrosis
through matrix stiffening and COX-2 suppression. J. Cell Biol..

[14] Cox T. R. (2021). The matrix
in cancer. Nat. Rev. Cancer.

[15] Wang W., Hapach L. A., Griggs L., Smart K., Wu Y., Taufalele P. V., Rowe M. M., Young K. M., Bates M. E., Johnson A. C. (2022). Diabetic hyperglycemia promotes primary tumor
progression through glycation-induced tumor extracellular matrix stiffening. Sci. Adv..

[16] Levental K. R., Yu H., Kass L., Lakins J. N., Egeblad M., Erler J. T., Fong S. F., Csiszar K., Giaccia A., Weninger W. (2009). Matrix
crosslinking forces tumor progression by enhancing integrin
signaling. Cell.

[17] Piersma B., Hayward M.-K., Weaver V. M. (2020). Fibrosis
and cancer: A strained relationship. Biochim.
Biophys. Acta, Rev. Cancer.

[18] Lampi M. C., Reinhart-King C. A. (2018). Targeting
extracellular matrix stiffness to attenuate
disease: From molecular mechanisms to clinical trials. Sci. Transl. Med..

[19] Bohrer M., Patterson G. D., Carroll P. (1984). Hindered diffusion of dextran and
ficoll in microporous membranes. Macromolecules.

[20] Fissell W. H., Manley S., Dubnisheva A., Glass J., Magistrelli J., Eldridge A. N., Fleischman A. J., Zydney A. L., Roy S. (2007). Ficoll is
not a rigid sphere. Am. J. Physiol. Renal Physiol..

[21] Venturoli D., Rippe B. (2005). Ficoll and dextran vs. globular proteins as probes for testing glomerular
permselectivity: effects of molecular size, shape, charge, and deformability. Am. J. Physiol. Renal Physiol..

[22] Ohlson M., Sorensson J., Haraldsson B. (2000). Glomerular size and charge selectivity
in the rat as revealed by FITC-Ficoll and albumin. Am. J. Physiol. Renal Physiol..

[23] Avendano A., Chang J. J., Cortes-Medina M. G., Seibel A. J., Admasu B. R., Boutelle C. M., Bushman A. R., Garg A. A., DeShetler C. M., Cole S. L. (2020). Integrated
biophysical characterization of fibrillar
collagen-based hydrogels. ACS Biomater. Sci.
Eng..

[24] Shaw M., Schy A. (1981). Diffusion coefficient
measurement by the″ stop-flow″
method in a 5% collagen gel. Biophys. J..

[25] Lazzara M. J., Deen W. M. (2004). Effects of concentration of the partitioning of macromolecules
mixtures in agarose gels. J. Colloid Interface
Sci..

[26] Tibbitt M. W., Anseth K. S. (2009). Hydrogels as extracellular matrix mimics for 3D cell
culture. Biotechnol. Bioeng..

[27] Caliari S. R., Burdick J. A. (2016). A practical guide
to hydrogels for cell culture. Nat. Methods.

[28] Fissell W.
H., Hofmann C. L., Smith R., Chen M. H. (2010). Size and conformation
of Ficoll as determined by size-exclusion chromatography followed
by multiangle light scattering. Am. J. Physiol.
Renal Physiol..

[29] Groszek J., Li L., Ferrell N., Smith R., Zorman C. A., Hofmann C. L., Roy S., Fissell W. H. (2010). Molecular conformation and filtration properties of
anionic Ficoll. Am. J. Physiol. Renal Physiol..

[30] Wang, D. ; Ferrell, N. In Vitro Models to Evaluate Molecular Permeability of the Kidney Filtration Barrier. Kidney Res. Exp. Protoc.; Springer, 2023; pp 41–53. 10.1007/978-1-0716-3179-9_4 37423981

[31] Oliver J., Anderson S., Troy J. L., Brenner B. M., Deen W. (1992). Determination of glomerular
size-selectivity in the normal rat with
Ficoll. Journal of the American Society of Nephrology.

[32] Ogston A.
G. (1958). The spaces
in a uniform random suspension of fibers. Trans.
Faraday Soc..

[33] Laurent T. C. (1967). Determination
of the structure of agarose gels by gel chromatography. Biochim. Biophys. Acta, Gen. Subj..

[34] Giddings J. C., Kucera E., Russell C. P., Myers M. N. (1968). Statistical theory
for the equilibrium distribution of rigid molecules in inert porous
networks. Exclusion chromatography. J. Phys.
Chem..

[35] Pluen A., Netti P. A., Jain R. K., Berk D. A. (1999). Diffusion of macromolecules
in agarose gels: comparison of linear and globular configurations. Biophys. J..

[36] Shaw M. L., Schy A. (1979). Molecular distribution within collagen
gel columns. J. Chromatogr. A.

[37] Laurent T. C., Killander J. (1964). A theory of
gel filtration and its exeperimental verification. J. Chromatogr. A.

[38] Ogston A. (1958). The spaces
in a uniform random suspension of fibres. Trans.
Faraday Soc..

[39] McCarty W. J., Johnson M. (2007). The hydraulic conductivity of Matrigel. Biorheology.

[40] Serwer P., Hayes S. J. (1986). Exclusion of spheres
by agarose gels during agarose
gel electrophoresis: dependence on the sphere’s radius and
the gel’s concentration. Anal. Biochem..

[41] Zhu B., Li W., Chi N., Lewis R. V., Osamor J., Wang R. (2017). Optimization
of glutaraldehyde vapor treatment for electrospun collagen/silk tissue
engineering scaffolds. ACS Omega.

[42] Olde
Damink L., Dijkstra P., Van Luyn M., Van Wachem P., Nieuwenhuis P., Feijen J. (1995). Glutaraldehyde as a crosslinking
agent for collagen-based biomaterials. J. Mater.
Sci.: Mater. Med..

[43] Perez-Puyana V., Romero A., Guerrero A. (2016). Influence of collagen concentration
and glutaraldehyde on collagen-based scaffold properties. J. Biomed. Mater. Res., Part A.

[44] Soltani F., Kaartinen M. T. (2023). Transglutaminases
in fibrosis—Overview and recent
advances. Am. J. Physiol. Cell Physiol..

[45] Grenard P., Bresson-Hadni S., El Alaoui S. d., Chevallier M., Vuitton D. A., Ricard-Blum S. (2001). Transglutaminase-mediated cross-linking
is involved in the stabilization of extracellular matrix in human
liver fibrosis. J. Hepatol..

[46] Paguirigan A. L., Beebe D. J. (2007). Protocol for the
fabrication of enzymatically crosslinked
gelatin microchannels for microfluidic cell culture. Nature protocols.

[47] Zhou J., Wu Y., Zhang X., Lai J., Li Y., Xing J., Teng L., Chen J. (2021). Enzyme catalyzed
hydrogel as versatile
bioadhesive for tissue wound hemostasis, bonding, and continuous repair. Biomacromolecules.

[48] Teixeira L. S. M., Feijen J., van Blitterswijk C. A., Dijkstra P. J., Karperien M. (2012). Enzyme-catalyzed
crosslinkable hydrogels: emerging strategies for tissue engineering. Biomaterials.

[49] Yung C., Wu L., Tullman J., Payne G., Bentley W., Barbari T. (2007). Transglutaminase
crosslinked gelatin as a tissue engineering scaffold. J. Biomed. Mater. Res., Part A.

[50] Lee P.-F., Bai Y., Smith R., Bayless K., Yeh A. (2013). Angiogenic responses
are enhanced in mechanically and microscopically characterized, microbial
transglutaminase crosslinked collagen matrices with increased stiffness. Acta Biomater..

[51] Yang Y., Ritchie A. C., Everitt N. M. (2017). Comparison
of glutaraldehyde and
procyanidin cross-linked scaffolds for soft tissue engineering. Mater. Sci. Eng., C.

[52] Mollenkopf P., Kochanowski J. A., Ren Y., Vining K. H., Janmey P. A., Purohit P. K. (2025). Poroelasticity and permeability of
fibrous polymer
networks under compression. Soft Matter.

